# Editorial: Vitiligo: from obscurity to spotlight – Advancing care with new therapies and AI

**DOI:** 10.3389/fmed.2026.1775222

**Published:** 2026-01-21

**Authors:** Yan Valle, Torello Lotti

**Affiliations:** 1 Vitiligo Research Foundation, New York, NY, United States; 2 Prisca Sapientia Institut, University Zurich, Zurich, Switzerland; 3 Istituto Superiore di Medicina, Florence, Italy

**Keywords:** artificial intelligence, digital health, global health, patient journey, quality of life, teledermatology, vitiligo, World Vitiligo Day

## Introduction

For a long time, vitiligo sat in what one might call a medical blind spot: too visible on the skin to ignore, yet strangely invisible in research budgets (1). It was easy to dismiss as “cosmetic,” to reassure patients with vague platitudes, and to move on to diseases that looked more serious on grant applications. Over the last decade, that posture has become increasingly untenable. If you follow the molecules, the data, and the patient stories—rather than the shifting social media optics—you see a field undergoing a genuine shift.

This Research Topic, “*Vitiligo: From Obscurity to Spotlight – Advancing Care with New Therapies and AI*,” was launched in September 2024 to capture that moment. Our aim as Topic Editors was not to curate a glossy showcase of the latest buzzwords but to bring together work that actually moves the needle: on therapies, on quality of life, on digital tools, and on the lived experience of people with vitiligo.

## From submissions to a curated Research Topic

Since launch, the Topic has attracted 21 submissions. After peer review, eight manuscripts were rejected, giving a rejection rate of approximately 40%. In other words, this Research Topic is not a transcript of everything that arrived in our inbox; it is a filtered snapshot of where evidence, methods, and clinical relevance aligned.

The 12 published articles can be grouped into five thematic clusters:

- AI and digital health (4 articles)
- Patient journey and care models (3 articles)
- Quality of life and sociodemographic burden (2 articles)
- Global advocacy and real-world impact (2 articles)
- Emerging regenerative approaches (1 article)

At the time of writing, these contributions have generated a total of 28,000 views and 4,800 downloads. For a disease that until recently struggled to get a single dedicated clinical trial funded, this level of engagement is not trivial.

## AI and digital health: beyond buzzwords

A prominent thread across the Topic is the maturation of digital and AI tools from conceptual promise toward clinical and operational relevance.

One original research contribution, “*Prototype of a Multimodal AI System for Vitiligo Detection and Mental Health Monitoring*,” integrates lesion detection with patient-reported psychological signals, illustrating a practical psychodermatology direction: measurement of skin status alongside structured monitoring of wellbeing (Biró et al.).

A complementary opinion article from Canada, “*Technological Advances in Vitiligo Management: Perspectives on AI, Mobile Tools, and Clinical Utility*,” takes a broader lens, surveying AI-driven diagnostics, mobile tools, and clinical decision-support systems (Parikh et al.). It is cautiously optimistic: new tools can extend the reach of dermatology but only if they are validated, explainable, and integrated into workflows rather than bolted on as gadgets.

The article “*Potential of Automated Image Analysis for the Measurement of Vitiligo Lesions*” from Italy moves the conversation down to the algorithmic fundamentals: segmentation, repeatability, and the elusive quest for standardization (Mazzetto et al.). It reminds us that good AI is 10% “intelligence” and 90% data hygiene and measurement discipline.

Finally, ”*Advancing Vitiligo Care in Russia: Landscape, Lessons, and a Scalable Digital Health Strategy*” proposes a coordinated national approach combining teledermatology, electronic records, and AI-enabled workflows to expand access across a geographically distributed population (Borodina et al.). The article also highlights locally developed therapeutics [including sodium acridone acetate (2)], illustrating how potentially useful regional practices can remain underrepresented internationally without the infrastructure and incentives required for broader evaluation.

More broadly, it is a reminder that some promising therapeutic approaches stay confined to regional practice—not because they lack merit but because international validation, multicenter trials, and regulatory pathways are costly and complex.

Across these contributions, the unifying question is not whether the technology can be built but whether it can be deployed in a way that improves access, measurement, continuity of care, and, ultimately, outcomes.

## Quality of life and patient experience: putting numbers to lived reality

Another strong axis of the Topic is the quantification and narration of the patient experience. Two original research articles from Romania—“*Comprehensive Analysis Of Life Quality Of Patients With Vitiligo in Romania: insights from a multivariate approach*” and “*Evaluation of the Clinical and Sociodemographic Features Of Patients With Vitiligo From The Central Region Of Romania*”—connect clinical variables to real-world burden, showing how disease extent, duration, and social context intersect to shape quality of life (Fekete, Bacârea et al., Fekete, Fekete et al.).

Complementing these quantitative views are three patient-centered opinion pieces.

- “*Understanding Vitiligo Through the Eyes of a Typical Patient in the U.S*.” walks the reader through what an “ordinary” journey actually feels like in a high-income healthcare system—delays, mixed messages, self-experimentation, and the constant negotiation between treatment fatigue and hope (Valle, Lotti, Sigova).

- “*Mapping the Vitiligo Patient Journey: From Awareness to Treatment or Coping Strategies*” zooms out to identify key transition points and decision nodes along that journey (Valle, Lotti, Towheed et al.).

- “*Vitiligo: a Call for Paradigm Shift Toward Comprehensive Patient Care*” argues for long-term planning that includes not only lesion-focused procedures but also comorbidity awareness and psychological support (Sigova et al.).

Together, these articles reinforce a practical point: improved therapies are necessary, but they are not sufficient if access, education, continuity, and psychosocial support remain inconsistent.

## Emerging therapeutics and regenerative approaches

Therapeutic innovation appears in this Topic through “*Injectable Crosslinked HA Hydrogel: A Promising Carrier for Cell Transplantation to Treat Stable Vitiligo*,” which illustrates how biomaterials may support regenerative and cell-based strategies in appropriately selected patients (Zheng et al.).

This contribution is also a reminder that vitiligo care is unlikely to converge on a single universal solution. Durable progress will likely be plural and combination-based—integrating topical agents, systemic therapies, phototherapy, surgery, and regenerative approaches in tailored regimens rather than relying on a single “one-size-fits-all” mechanism.

## World Vitiligo Day and the politics of visibility

Vitiligo does not exist in a social vacuum, and neither does vitiligo research. Two opinion articles examine World Vitiligo Day (WVD) as both a patient-led movement and a mechanism for sustained change (3). “*World Vitiligo Day: A Model for Grassroots Medical Activism and Pharmaceutical Innovation*” describes how awareness can translate into research attention and industry engagement (Valle, Arenas Soto, et al.). “*World Vitiligo Day: Lessons from Mexico's Annual Headquarters and Its Real-World Impact*” provides a longitudinal country case study of how consistent national involvement can strengthen patient education, specialized services, and local ecosystems (Ocampo-Candiani et al.).

The broader implication is that visibility matters most when it builds durable structures—registries, centers of expertise, patient organizations, and reproducible care pathways.

## A unifying framework

Across themes, three messages recur.

First, vitiligo is increasingly understood as a chronic, immune-mediated disease with meaningful clinical and psychosocial consequences, requiring longitudinal care models rather than episodic, lesion-only interventions.

Second, AI and digital tools are advancing rapidly, but their value depends on validation, measurement discipline, and thoughtful implementation, not terminology.

Third, the greatest impact emerges at the intersection of therapies, digital infrastructure, and patient-centered systems: effective treatments should reach patients through pathways shaped by accurate information, appropriate triage and monitoring, and supportive communities.

This can be conceptualized as a three-layer framework: (i) therapies and regenerative approaches, (ii) digital and AI infrastructure, and (iii) patient journey and advocacy. Where these layers overlap lies the practical goal of durable and equitable improvement in outcomes for people living with vitiligo.

## Looking ahead

As Topic Editors, we are grateful to the authors, reviewers, and readers who contributed to this Research Topic. Our hope is that “*Vitiligo: From Obscurity to Spotlight – Advancing Care with New Therapies and AI*” will serve not only as a record of where the field stands today but as a reminder of what still needs to be done.

Vitiligo has entered the spotlight. The next task is to ensure that increased attention translates into clarity—about therapies, about tools, and about the care structures that enable people with vitiligo not merely to cope but to live well.
